# Winter Night-Warming Improves Post-anthesis Physiological Activities and Sink Strength in Relation to Grain Filling in Winter Wheat (*Triticum aestivum* L.)

**DOI:** 10.3389/fpls.2017.00992

**Published:** 2017-06-13

**Authors:** Yonghui Fan, Zhongwei Tian, Yanyan Yan, Chenxi Hu, Muhammad Abid, Dong Jiang, Chuanxi Ma, Zhenglai Huang, Tingbo Dai

**Affiliations:** ^1^Key Laboratory of Crop Physiology, Ecology and Production Management, Nanjing Agricultural UniversityNanjing, China; ^2^School of Agronomy, Anhui Agricultural UniversityHefei, China

**Keywords:** night-warming, grain filling, photosynthesis, source-sink strength, winter wheat (*Triticum aestivum* L.)

## Abstract

The diurnal and seasonal temperature rising patterns “asymmetric warming,” plays an important role in crop distribution and productivity. Asymmetric warming during the early growth periods of winter wheat (*Triticum aestivum* L.) profoundly affects vegetative growth and post-anthesis grain productivity, but the underlying physiological mechanism is still unclear. We conducted field experiments from 2012 to 2014 using two wheat cultivars, namely, Yangmai-13 (vernal type) and Yannong-19 (semi-winter type), to investigate the influences of night-warming during the winter (warming by 1.56–1.67°C from tillering to jointing) or during the spring (warming by 1.78–1.92°C from jointing to booting) on post-anthesis physiological activities and grain-filling processes. Both night-warming treatments enhanced the source activity by increasing flag leaf area, chlorophyll content, and photosynthetic capability in both cultivars compared with those of the control. The night-warming treatments caused an increase in the antioxidant activities of superoxide dismutase (SOD), peroxidase, and catalase (CAT) in the flag leaves of both cultivars, while ROS contents such as superoxide anion radical ($O_{2}^{•-}$) and hydrogen peroxide (H_2_O_2_) decreased. Moreover, the expression levels of Rubisco activase B (*RcaB*), major chlorophyll a/b-binding protein (*Cab*), chloroplast Cu/Zn superoxide dismutase (*Cu/Zn-SOD*), mitochondrial manganese superoxide dismutase (*Mn-SOD*), and *CAT* genes were upregulated at anthesis and were associated with higher photosynthetic capacity and antioxidant activities. Furthermore, night-warming improved sink activities by increasing the concentrations of grain indole-3-acetic acid and cytokinins as well as the sucrose synthase activity for both cultivars. Winter night-warming showed greater potential for improving source strength and grain filling, with consistent performance in both cultivars compared with that of spring night-warming. We concluded form these results that night-warming can improve source and sink capacities in winter wheat, and winter night-warming has greater advantages in this respect than does spring warming.

## Introduction

Temperature affects the development, growth, and physiological processes of plants (Asseng et al., 2013). The air temperature of the earth experienced significant increases during the 1950s–2000s (Intergovernmental Panel on Climate Change [IPCC], 2007). Global warming shows variable diurnal and seasonal warming patterns known as asymmetric warming. Increases of temperature amplitude in winter and spring are greater than in autumn and summer. Also, warming is greater at night than during the day, which describes a phenomenon commonly referred to as asymmetric warming (Intergovernmental Panel on Climate Change [IPCC], 1998; Su et al., 2015). These climatic trends are likely to further increase until 2050 (Chavas et al., 2009). Winter wheat is normally cultivated over the winter and spring seasons (Liu et al., 2014). As a warm- and cool-season crop, this sort of climate warming is likely to profoundly influence winter wheat. However, experiments simulating the impact of warming on winter wheat are mostly based on mean air temperature, without consideration for diurnal warming asymmetry.

Warming was reported to have dramatic effects on the morpho-physiological processes of plants, including altered crop development and growth (Tao and Zhang, 2013), diminished functionality of photosystem II (PSII) (Atkin and Tjoelker, 2003), and alleviated antioxidant systems (Martinez et al., 2014), and to influence the duration of grain filling (Liu Z. et al., 2013). An increase in night temperature by 1.1°C promotes grain-filling rates both in superior and inferior grains, ultimately resulting in a significantly increased 1,000-grain weight in wheat (Chen et al., 2014). In a 5-year experiment, it is found that the grain yield of winter wheat increased by 16.3%, and the grain-filling rate of inferior grain was stimulated by a full-day temperature increase of 1.5°C (Tian et al., 2012). Plant hormones are key regulators of grain development under different plant growth conditions (Yang et al., 2006). Morris et al. (1993) observed a large transient rise in cytokinin (CTK) levels after anthesis in the developing grains of wheat and rice that significantly affected the duration of grain filling and grain size. Lur and Setter (1993) reported that indole-3-acetic acid (IAA) content rapidly increased in maize (*Zea mays*) kernels at nearly 10 days after anthesis, which resulted in an increase in assimilate transport to the developing kernels and ultimately resulted in higher kernel weights.

Crop yield is closely related to net photosynthetic assimilation process during the grain-filling period in wheat. The photosynthesis of the flag leaf contributes the most to final grain dry weight in winter wheat. Study of the flag leaf in response to climate warming that occurs during the winter and spring seasons is crucial. Photosynthesis is the most temperature-sensitive process in plants. Some species can adapt to temperature change, as indicated by shifts in the thermal optimum and improved photosynthetic rates at new growth temperatures (Chen et al., 2014). Martinez et al. (2014) found that when temperature increases to the optimal range of the plant, the photosynthetic rate is expected to increase. However, the mechanism of temperature acclimation regarding photosynthesis is still unclear. Some studies assumed that this acclimation is primarily due to the increased regeneration capability of ribulose-1,5-bisphosphate (RuBP) and the increased carboxylation capacity of Rubisco (Wang et al., 2011). According to the models of Farquhar et al. (1980), the balance between RuBP regeneration and carboxylation, as indicated by the maximum rate of carboxylation (*V*_cmax_) versus the maximum rate of photosynthetic electron transport (*J*), determines the temperature dependence of photosynthesis. However, evidence from previous experiments does not clarify the mechanism of photosynthetic change under climate warming during the entire life cycle of winter wheat.

Photosynthetic properties and senescence in plants are inherently programmed, but both are influenced largely by environmental conditions (Riikonen et al., 2009). Lobell et al. (2012) suggested that chlorophyll content could determine the photosynthetic capacity of plants, which has a positive correlation with the chloroplast membrane stability, and could be used to screen for heat stress in winter wheat. Reactive oxygen species (ROS), which are involved in the physiological metabolism and life processes of plants, inhibit photosynthesis. To prevent ROS accumulation, plants have developed certain antioxidant systems. The effects of heat stress on membrane peroxidation, reduction of PSII activity, and chlorophyll loss are caused by oxidative stress related to overproduction of ROS (Djanaguiraman et al., 2010). Most of the studies on both thermal acclimation and the characteristics of oxidative metabolism have been conducted using constant temperature regimes and have occurred in greenhouse or growth chambers (Atkin and Tjoelker, 2003; Xu et al., 2008). However, how antioxidant capacities and the production of ROS respond to asymmetric warming conditions under field conditions remains to be determined.

It has been shown that abiotic stress priming or adaptation during vegetative periods can improve plant stress tolerance during subsequent reproductive growth stages (Cui et al., 2015). For example, winter wheat subcellular antioxidant systems were activated under cold priming, and consequently, the tolerance to subsequent cold stress was enhanced (Li et al., 2014a). Pre-anthesis high-temperature priming enhanced plant photosynthesis due to the expression of the photosynthesis-responsive gene *RcaB* (encoding Rubisco activase B) and the major chlorophyll a/b-binding protein gene *Cab* (Wang et al., 2011). Li et al. (2014b) found that the expression of antioxidant enzyme-related genes such as *Cu/Zn-SOD*, which encodes chloroplast Cu/Zn superoxide dismutase, and *Mn-SOD*, which encodes mitochondrial manganese superoxide dismutase, was upregulated under drought acclimation. Heat priming using a moderate temperature can improve tolerance to subsequent heat stress in plants (Larkindale and Huang, 2004). However, it is still unclear if asymmetrical warming during vegetative periods can improve the post-anthesis physiological activities of winter wheat.

We have reported that winter night-warming promoted pre-anthesis plant growth and improved post-anthesis net photosynthetic rates, ultimately increasing grain yield (Fan et al., 2015). In the present study, winter wheat plants were exposed to night-warming during the winter and spring seasons to examine grain-filling processes, grain sucrose content, grain endogenous hormones, photosynthetic capacity, antioxidant capacity, the expression of photosynthesis-responsive genes, and antioxidant enzyme-related genes during the reproductive growth period. Our main objective was to determine whether winter and spring night-warming could improve the post-anthesis source activity and sink strength of winter wheat and to clarify the possible physiological mechanisms involved. We hope the results will contribute to better understanding of the physiological mechanisms causing wheat yield increase under winter and spring night-warming.

## Materials and Methods

### Experimental Site

The experiments were conducted from 2012 to 2014 in a humid, semi-tropical region in Nanjing (32°04′ N, 118°76′ E), Jiangsu Province, China. The mean annual temperature was 15°C, the annual rainfall was approximately 1000 mm, and the incoming solar radiation was 4530 MJ m^-2^ y^-1^. The 0-20 cm soil depth contained 10.95 g kg^-1^ of organic matter, 0.79 g kg^-1^ of available N, 9.85 mg kg^-1^ of Olsen-P, and 72.30 mg kg^-1^ of K_2_O.

### Field Experimental Design

Two warming treatments: the winter night-warming treatment (WW), spring night-warming treatment (SW), with no warming as the control group (NW). The experiment was a split-plot design, with warming as the main plot and wheat cultivar as the subplots, and consisted of three replicates each.

**FIGURE 1:**
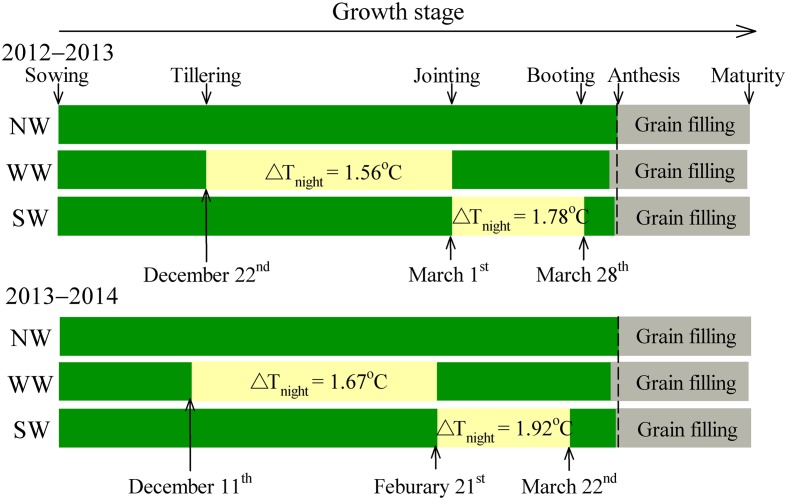
Schematic representation of experimental design and treatments. WW and SW refer to night-warming during the winter and spring seasons, respectively. NW refers to the no warming control. ΔT_night_ refers to the increase in mean night temperature between treatments and the control. Mean night temperature is the mean in all temperature data on a 10-min interval from 19:00 to 07:00 h.

The warming treatment was based on the technique of passive night-warming; the treatment details are described by Fan et al. (2015) and thus are only briefly introduced here. The warmed plot was covered with a plastic membrane from 19:00 h to 07:00 h of the next day. The warming facility was 3 m in width, 5 m in length, and 2 m in height. The monthly precipitation and climate data are described by Fan et al. (2015). The experimental design is shown in **Figure 1**.

### Crop Management

Two winter wheat cultivars, namely, Yangmai-13 (vernal type) and Yannong-19 (semi-winter type), were used. The sowing dates were November 2, 2012 and October 26, 2013, respectively, at a seeding rate of 180 plants m^-2^, with a 25-cm row spacing both years. The experiments in 2 years were performed at different sites, where wheat cropping followed the maize season in a wheat-maize rotation. The plots were 2 m × 4 m in size. Each year, doses of 120 kg N hm^-2^, 105 kg P_2_O_5_ hm^-2^, and 150 kg K_2_O hm^-2^ were applied before sowing, and another 120 kg N hm^-2^ was applied as a top-dressing at the jointing and booting stages. N fertilizer was added in the form of urea (46% N). We used the same irrigation regime to ensure that wheat growth occurred without water limitation throughout the experimental period for all of the plots (and only when irrigation was necessary based on soil moisture conditions).

### Sampling Method and Physiological Measurements

Uniform tillers flowering on the same day were tagged for sampling and measurements. We sampled 20 tagged spikes from three plots per treatment at intervals of 7 days until maturity from anthesis. Flag leaves and grain were detached immediately, and all flag leaves and half of the grain were frozen in liquid nitrogen for 2 h and then stored at –80°C. The other half of the grain was dried in an oven at 80°C until constant weight for measuring the grain dry weight and grain sucrose content. The grain-filling rate was estimated according to the methods of Chen et al. (2014). We measured three replicates of each treatment.

#### Grain Sucrose Content, Grain Sucrose Synthase (SS, EC 2.4.1.13) Activity, Indole-3-Acetic Acid (IAA) Concentration, and Cytokinin (CTK) Concentration

The sucrose content and SS activity were determined according to the methods of Ahmadi and Baker (2001). Endogenous hormones were analyzed using an enzyme-linked immunosorbent assay (ELISA) according to the methods of Han et al. (2015). All of the reagents used in the ELISA were purchased from the Phytohormones Research Institute of the Chinese Agricultural University (Beijing, China).

#### Chlorophyll Content, Net Photosynthetic Rate (Pn) and Calculation of the Response to Substomatal CO_2_ Concentration

The chlorophyll content was determined according to the methods of Arnon (1949). We measured the Pn of flag leaves (five tagged leaves per plot) on a sunny day between 09:30 and 11:00 h at 0 and 14 days after anthesis (DAA), using an LI-6400 portable photosynthesis measurement device (Li-Cor Inc., United States). The chamber was equipped with a red/blue LED light source (LI6400-02B). Photosynthetic measurements were performed under light-saturated conditions (1000 μmol photons m^-1^ s^-1^ of PPFD) at 25°C and 400 μmol CO_2_ mol^-1^ (Ca). We also determined net CO_2_ assimilation rate (*A/Ci*) curves using the LI-6400 portable photosynthesis system at 0 and 14 DAA for light-adapted flag leaves. Prior to measurements, the leaves were placed in the chamber at a photosynthetically active radiation (PAR) level of 1200 μmol m^-2^ s^-1^, and the CO_2_ concentration in the chamber was set at 380 μmol CO_2_ mol^-1^. Ten minutes later, the responses of the net carbon assimilation rate versus the intercellular CO_2_ concentration were determined at CO_2_ concentrations of 380, 200, 150, 100, 50, 400, 600, 800, 1000, 1200, and 1500 μmol CO_2_ mol^-1^. The maximum photosynthetic rate (*A*_sat_), the maximum carboxylation rate (*V*_cmax_) of Rubisco, and the maximum rate of photosynthetic electron transport (*J*) were determined according to the methods of Sharkey et al. (2007).

#### Superoxide Anion Radical ($O_{2}^{•-}$) Production Rate, Hydrogen Peroxide (H_2_O_2_) Content, Malondialdehyde (MDA) Content, and Antioxidant Enzyme Activity

The $O_{2}^{•-}$ production rate was determined according to the methods of Sui et al. (2007). The H_2_O_2_ content and MDA content were determined according to the methods of Zheng et al. (2009). Superoxide dismutase (EC 1.15.1.1) activity was measured according to the methods of Yang et al. (2007), and peroxidase (POD, EC 1.11.1.7) activity was determined according to the methods of Zheng et al. (2009). Catalase (CAT, EC 1.11.1.6) activity was determined according to the methods of Tan et al. (2008).

#### Real-Time Quantitative PCR (qPCR) Analysis of Photosynthesis-Responsive Genes and Antioxidant Enzyme-Related Gene Expression

We analyzed the expression levels of genes encoding Rubisco activase B (*RcaB*, accession No. AF251264), the major chlorophyll a/b-binding protein (*Cab*, accession No. M10144), chloroplast Cu/Zn superoxide dismutase (*Cu/Zn-SOD*, accession No. U69632), mitochondrial manganese superoxide dismutase (*Mn-SOD*, accession No. AF092524), and catalase (*CAT*, accession No. D86327) using qPCR. The expression levels of these genes were determined according to the procedure described by Wang et al. (2011). To normalize the results, the relative abundance of *Actin* was determined and used as an internal standard.

Total RNA was isolated from wheat leaves (100 mg of fresh weight) at anthesis using Trizol reagent (Invitrogen, United States) following the manufacturer’s protocol. RNA integrity was verified electrophoretically using ethidium bromide staining and via an OD_260_/OD_280_-nm absorption ratio greater than 1.95. DNA-free total RNA (5 mg) from the various treatments was used with a PrimeScript RT reagent kit with gDNA Eraser (Takara, Japan). The cDNA reaction mixture was diluted to 1:20 using ddH_2_O for quantitative real-time PCR (qRT-PCR).

Quantitative real-time PCR was conducted using SYBR^®^ *Premix Ex Taq*^TM^ (TaKaRa, Japan) and the CFX96 Touch Real-Time PCR Detection System (Bio-Rad, United States). Each reaction was run in triplicate and contained 12.5 μL of SYBR *Premix Ex Taq*, 1.0 μL of each primer (10 μM), 2 μL of cDNA template, and 8.5 μL of ddH_2_O to form a final reaction volume of 25 μL. The cycling conditions were 95°C for 30 s followed by 40 cycles of 95°C for 5 s, 60°C for 31 s, and 72°C for 20 s. A melting curve (65–95°C, at increments of 1.0°C) was generated to check the specificity of amplification. Samples were also run on a 3% agarose gel to confirm specificity. The quantification cycle value (*C*q) and the appropriate standard curve were used to calculate the relative expression level of each target gene and the relative expression level of the housekeeping gene (*Actin*) in each sample. Relative fold differences were calculated based on the comparative *C*q method using *Actin* as an endogenous control. To determine the relative differences for each target gene in each treatment, the *C*q value for the target gene was normalized to the *C*q value for *Actin* and was calculated relative to a calibrator using the formula:

$$2^{-\Delta \Delta Cq}=2^{\left[-\Delta Cq(\text{sample})-\Delta Cq\left(\text{calibrator}\right)\right]}=2^{-\left[(\Delta Cq(\text{sample})-\Delta Cq\left(\text{Actin}\right)-\left(Cq(\text{calibrator})-Cq\left(\text{Actin}\right)\right)\right]}$$

Three biological replicates were used for each treatment.

### Statistical Analysis

A one-way ANOVA was performed on flag leaf area, specific leaf weight, chlorophyll content, $O_{2}^{•-}$ production rate, H_2_O_2_ content, MDA content, antioxidative enzyme activity, gene expression, sucrose content, SS activity, and endogenous hormone concentrations to determine the significant differences between the treatments at each time point. A two-way analysis of variation (ANOVA) was performed on Pn, *A*_sat_, *V*_cmax_, *J*, and grain-filling rate to determine the significant differences among cultivars and warming treatments. Statistical analyses were conducted using SPSS statistical software (SPSS ver. 10, SPSS, Chicago, IL, United States).

## Results

### Flag Leaf Morphology

**FIGURE 2:**
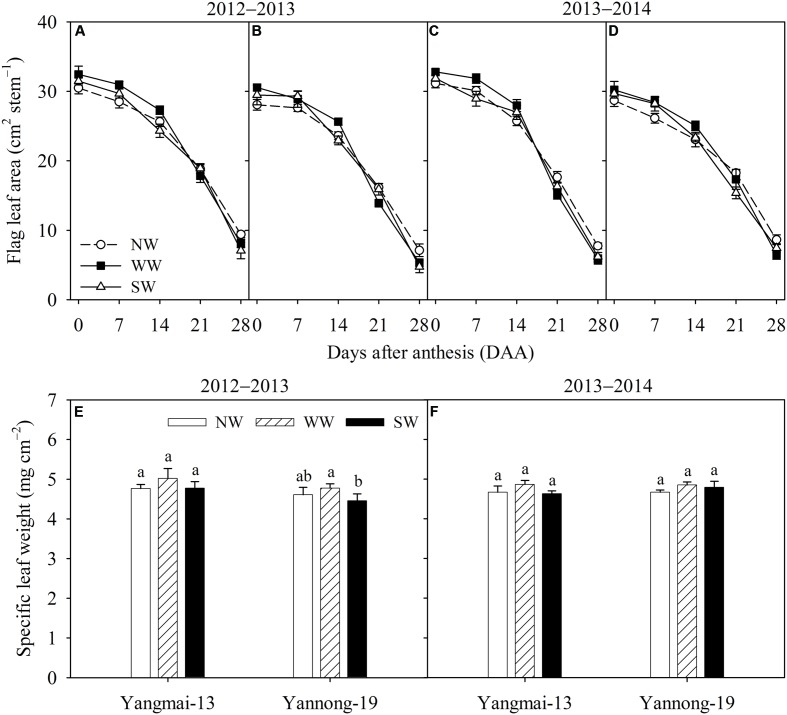
Flag leaf area **(A–D)** and flag leaf specific leaf weight **(E,F)** of Yangmai-13 and Yannong-19 after anthesis as affected by winter (WW) and spring (SW) night-warming during 2012–2013 and 2013–2014. NW refers to the no warming control. The data are the means ± SE (*n* = 3). Lowercase letters refer to significant differences between treatments (*P* < 0.05). Whiskers on the top of the bars indicate the standard error.

During the 2012–2013 season, the flag leaf areas of Yangmai-13 and Yannong-19 were higher for WW than for the control from 0 to 14 DAA, while the flag leaf area increased significantly at 7 and 14 DAA in Yangmai-13 and at 0 and 14 DAA in Yannong-19 (**Figures 2A,B**). During the 2013–2014 season, WW significantly increased the flag leaf area during early grain-filling stage (0-14 DAA) for both cultivars (**Figures 2C,D**). SW significantly increased the flag leaf area of Yannong-19 only at 7 DAA (**Figure 2D**). The flag leaf area was lower for WW and SW than for the control from 21 to 28 DAA (**Figures 2A–D**). At 14 DAA, WW slightly increased flag leaf specific leaf weight of both cultivars, but the effects were not significant (**Figures 2E,F**). ST had no effect on flag leaf specific leaf weight.

### Chlorophyll Content

**FIGURE 3:**
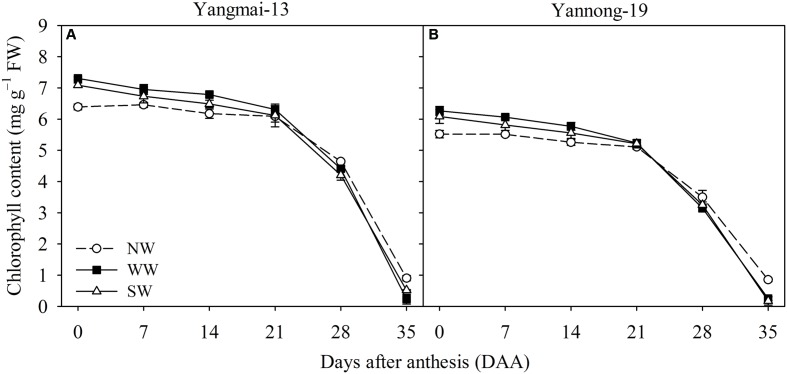
Chlorophyll content as affected by winter (WW) and spring (SW) night-warming in the flag leaves of Yangmai-13 **(A)** and Yannong-19 **(B)** after anthesis during 2013–2014. NW refers to the no warming control. The data are the means ± SE (*n* = 3).

From 0 to 14 DAA, WW and SW increased the flag leaf chlorophyll content of Yangmai-13 and Yannong-19, and the chlorophyll content was lower for WW and SW than for the control from 28 to 35 DAA (**Figure 3**). For Yangmai-13, WW significantly increased flag leaf chlorophyll content at 0 and 14 DAA, while SW significantly increased the chlorophyll content only at 0 DAA (**Figure 3A**). In Yannong-19, WW significantly increased flag leaf chlorophyll content during the early grain-filling stage (0-14 DAA) more than the control did, and SW significantly increased the chlorophyll content at 0 and 7 DAA (**Figure 3B**).

### Photosynthetic Capacity

At anthesis and the grain-filling stage, WW significantly increased the Pn of Yangmai-13 and Yannong-19, and SW significantly increased the Pn at the grain-filling stage of Yangmai-13 (**Table 1**). WW significantly increased the *A*_sat_ in flag leaves at anthesis and at the grain-filling stage of Yangmai-13, while SW significantly increased the *A*_sat_ of Yannong-19 at the grain-filling stage. WW significantly increased *V*_cmax_ in the flag leaves at anthesis and at the grain-filling stage for both cultivars, while WW surpassed SW in increasing *V*_cmax_ compared with the control. For Yangmai-13, WW and SW significantly increased *J* in flag leaves at anthesis and in the grain-filling stage, and the increases were higher for WW than for SW. In Yannong-19, WW significantly increased *J* at anthesis and at the grain-filling stage as compared with the control.

**Table 1:** Net photosynthetic rate (Pn, μmol CO_2_ m^-2^ s^-1^), maximum photosynthetic rate (*A*_sat_, μmol m^-2^ s^-1^), maximum carboxylation rate (*V*_cmax_, mol m^-2^ s^-1^), and maximum rate of photosynthetic electron transport (*J*, mol mol^-1^) of flag leaves at anthesis and grain-filling stage as affected by winter (WW) and spring (SW) night-warming during 2013–2014.

Cultivar	Treatment	Anthesis				Grain filling			
			
		Pn	*A*_sat_	*V*_cmax_	*J*	Pn	*A*_sat_	*V*_cmax_	*J*
Yangmai-13	NW	23.85 b	26.23 b	62.80 b	160.33 b	23.48 b	24.94 b	54.21 b	127.11 b
	WW	26.81 a	29.06 a	68.32 a	165.57 a	26.20 a	26.84 a	58.20 a	129.76 a
	SW	25.89 ab	27.10 ab	68.18 a	167.03 a	25.58 a	26.41 a	56.45 ab	124.42 c
Yannong-19	NW	23.81 b	25.58 a	63.27 b	122.39 b	23.49 b	23.53 c	47.02 b	110.25 ab
	WW	26.30 a	27.22 a	67.19 a	126.89 a	25.64 a	26.61 a	53.48 a	112.54 a
	SW	25.50 ab	26.84 a	64.06 ab	124.61 ab	23.62 b	24.22 bc	48.96 b	107.90 b


*NW refers to the no warming control. The data are the means ± SE (*n* = 3). Lowercase letters following the data within the same column refer to significant differences (*P* < 0.05).*

### $O_{2}^{•-}$ Production Rate, H_2_O_2_ Content, and MDA Content

**FIGURE 4:**
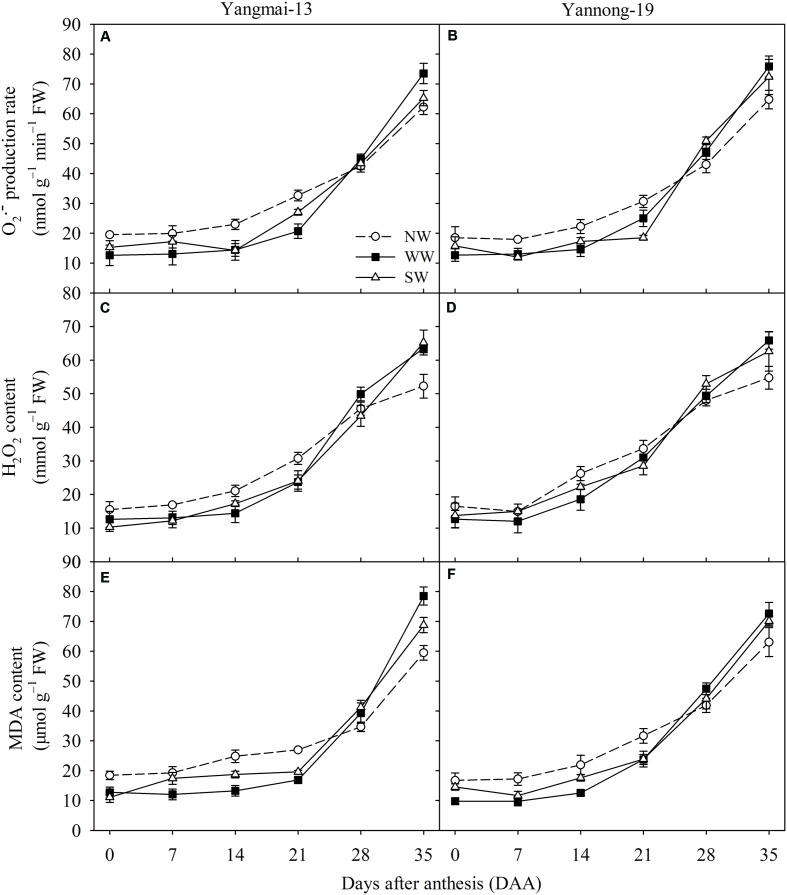
Superoxide anion radical ($O_{2}^{•-}$) production rate, hydrogen peroxide (H_2_O_2_) content, and malondialdehyde (MDA) content as affected by winter (WW) and spring (SW) night-warming in the flag leaves of Yangmai-13 **(A,C,E)** and Yannong-19 **(B,D,F)** after anthesis during 2013–2014. NW refers to the no warming control. The data are the means ± SE (*n* = 3).

WW and SW decreased the flag leaf $O_{2}^{•-}$ production rate of Yangmai-13 and Yannong-19 from 0 to 21 DAA (**Figures 4A,B**). For Yangmai-13, WW significantly reduced the $O_{2}^{•-}$ production rate at 0, 14, and 21 DAA; SW significantly decreased at 14 and 21 DAA (**Figure 4A**). For Yannong-19, WW significantly reduced the $O_{2}^{•-}$ production rate from 0 to 21 DAA; SW significantly decreased from 7 to 21 DAA (**Figure 4B**). The flag leaf H_2_O_2_ contents were lower under the WW and SW treatments than under NW from 0 to 21 DAA for Yangmai-13 and Yannong-19, while the H_2_O_2_ contents were higher for the WW and SW treatments than for the NW during the later stage of grain filling (28-35 DAA) (**Figures 4C,D**). Compared with NW, WW, and SW reduced the flag leaf MDA content of Yangmai-13 and Yannong-19 from 0 to 21 DAA, while WW significantly reduced the flag leaf MDA content (**Figures 4E,F**). The MDA content was higher under WW and SW than under NW during the later stage of grain filling (28-35 DAA).

### Activities of Antioxidative Enzymes

**FIGURE 5:**
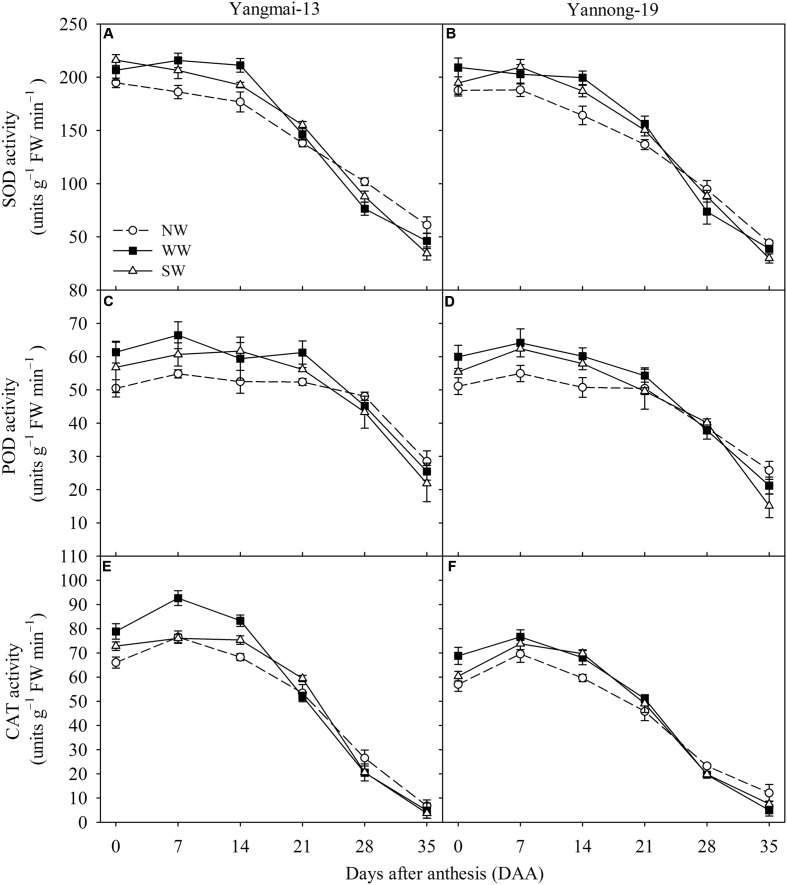
Activities of superoxide dismutase (SOD), peroxidase (POD), and catalase (CAT) as affected by winter (WW) and spring (SW) night-warming in the flag leaves of Yangmai-13 **(A,C,E)** and Yannong-19 **(B,D,F)** after anthesis during 2013–2014. NW refers to the no warming control. The data are the means ± SE (*n* = 3).

WW and SW increased the flag leaf SOD activity from 0 to 21 DAA for Yangmai-13 and Yannong-19, while WW and SW reduced SOD activity from 28 to 35 DAA compared with NW (**Figures 5A,B**). For Yangmai-13, WW and SW increased the flag leaf POD activity from 0 to 21 DAA compared with the NW, and the increases were greater for WW than for SW at 0, 7, and 21 DAA (**Figure 5C**). For Yannong-19, WW and SW increased POD activity from 0 to 21 DAA and from 0 to 14 DAA, respectively (**Figure 5D**). Compared with NW, WW significantly increased flag leaf CAT activity from 0 to 14 DAA for both cultivars (**Figures 5E,F**), while SW significantly increased the flag leaf CAT activity at 0 and 14 DAA in Yangmai-13 (**Figure 5E**) and at 14 DAA in Yannong-19 (**Figure 5F**). This indicated that night-warming during the winter and spring enhanced post-anthesis antioxidant capacity in the flag leaves of winter wheat.

### Photosynthesis-Responsive Gene Expression

**FIGURE 6:**
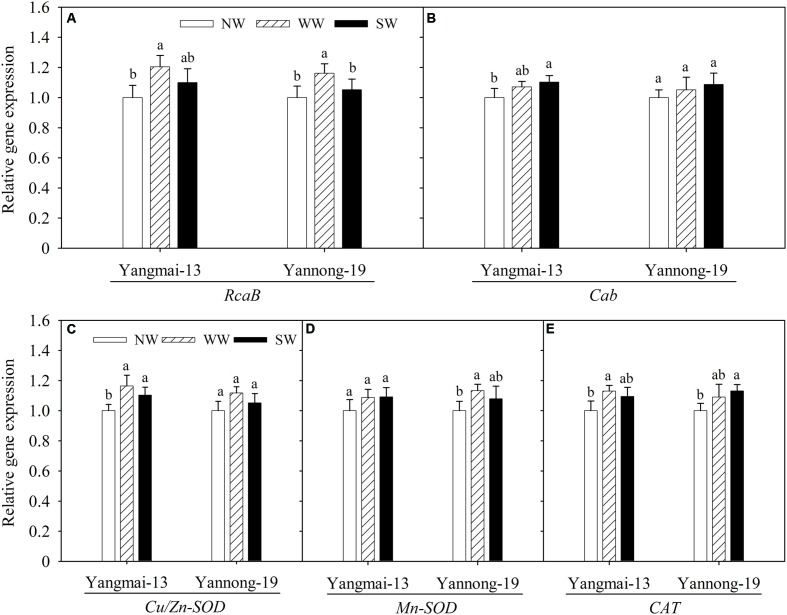
Relative expression of *RcaB* **(A)**, *Cab* **(B)**, *Cu/Zn-SOD* **(C)**, *Mn-SOD* **(D)**, and *CAT* **(E)** as affected by winter (WW) and spring (SW) night-warming in the flag leaves of Yangmai-13 and Yannong-19 at anthesis during 2013–2014. NW refers to the no warming control. *RcaB*, encode the Rubisco activase B; *Cab*, encode the major chlorophyll a/b-binding protein; *Cu/Zn-SOD*, encode chloroplastic Cu/Zn superoxide dismutase; *Mn-SOD*, encode the mitochondrial manganese superoxide dismutase; *CAT*, encode catalase. Lowercase letters refer to significant differences between treatments (*P* < 0.05). Whiskers on the top of the bars indicate the standard error (*n* = 3).

The photosynthesis-responsive genes analyzed here included *RcaB* and *Cab*. The WW treatment upregulated *RcaB* expression in the flag leaves of both cultivars (**Figure 6A**). The influences of WW and SW on *Cab* expression were not significant, except for Yangmai-13 under SW (**Figure 6B**).

### Antioxidant Enzyme-Related Gene Expression

Both WW and SW upregulated *Cu/Zn-SOD* expression in the flag leaves of Yangmai-13 compared with NW, and the extent of the upregulated expression of *Cu/Zn-SOD* was higher for WW than for SW (**Figure 6C**). In Yangmai-13, the effects of WW and SW on *Mn-SOD* expression in the flag leaves were not significant (**Figure 6D**). In Yannong-19, *Mn-SOD* expression was upregulated by WW. In addition, WW upregulated *CAT* expression more than NW did in Yangmai-13, while SW upregulated *CAT* expression in Yannong-19 (**Figure 6E**).

### Sucrose Content and SS Activity

**FIGURE 7:**
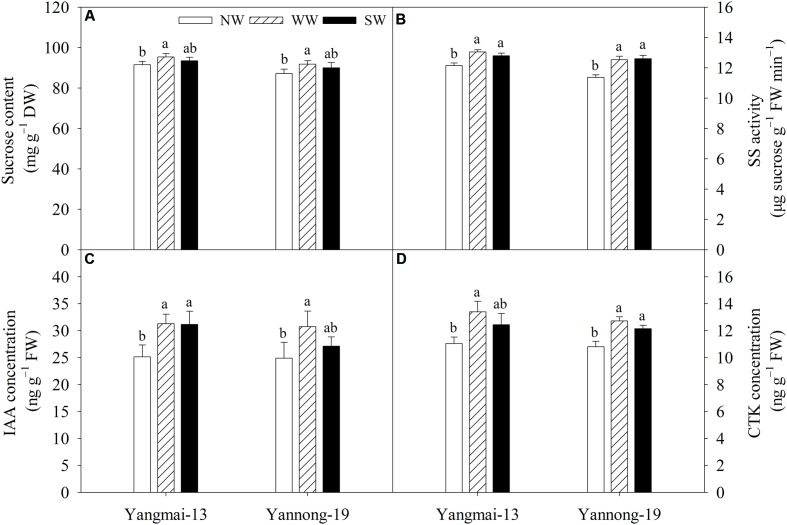
Grain sucrose content **(A)**, sucrose synthase (SS) activity **(B)**, indole-3-acetic acid (IAA) concentration **(C)** and cytokinin (CTK) concentration **(D)** of Yangmai-13 and Yannong-19 at the grain-filling stage as affected by winter (WW) and spring (SW) night-warming during 2013–2014. NW refers to the no warming control. Lowercase letters refer to significant differences between treatments (*P* < 0.05). Whiskers on the top of the bars indicate the standard error (*n* = 3).

WW significantly increased the grain sucrose content at the grain-filling stage in both cultivars, but there was no effect on grain sucrose content under SW (**Figure 7A**). WW and SW significantly increased grain SS activity at the grain-filling stage in both cultivars (**Figure 7B**).

### IAA and CTK Concentration

WW significantly increased the grain IAA concentration at the grain-filling stage for both cultivars, while SW significantly increased the grain IAA concentration of only Yangmai-13 (**Figure 7C**). WW significantly increased the grain CTK concentration at the grain-filling stage for both cultivars, while SW significantly increased grain CTK concentration of only Yannong-19 (**Figure 7D**).

### Grain-Filling Rate

The post-anthesis grain-filling rates were higher for WW and SW than for NW in both cultivars (**Table 2**). The higher grain-filling rate was found at the early stage (7-21 DAA). WW showed greater potential for improving the grain-filling duration than did SW.

**Table 2:** Grain-filling rate of Yangmai-13 and Yannong-19 as affected by winter (WW) and spring (SW) night-warming during 2012-2014.

Treatment		Grain-filling rate (mg grain^-1^ d^-1^)				
		
		0-7 DAA	7-14 DAA	14-21 DAA	21-28 DAA	28-Maturity
**2012–2013**						
Yangmai-13	NW	0.94 a	1.44 bc	1.60 b	1.40 b	1.06 c
	WW	0.99 a	1.57 a	1.69 a	1.47 a	1.03 d
	SW	0.98 a	1.49 ab	1.65 a	1.45 a	1.04 cd
Yannong-19	NW	0.88 a	1.35 c	1.50 c	1.31 c	1.14 a
	WW	0.94 a	1.44 bc	1.59 b	1.39 b	1.11 b
	SW	0.90 a	1.38 bc	1.53 c	1.34 c	1.10 b
**2013–2014**						
Yangmai-13	NW	0.95 ab	1.48 b	1.65 bc	1.40 bc	1.12 bcd
	WW	1.03 a	1.60 a	1.73 a	1.46 a	1.08 d
	SW	0.98 ab	1.53 ab	1.70 ab	1.44 ab	1.10 cd
Yannong-19	NW	0.84 b	1.40 c	1.55 d	1.35 c	1.18 a
	WW	0.87 b	1.49 b	1.63 bc	1.42 ab	1.13 bc
	SW	0.87 b	1.45 bc	1.61 cd	1.40 bc	1.16 ab


*NW refers to the no warming control. DAA refers to days after anthesis. The data are the means ± SE (*n* = 3). Lowercase letters following the data within the same column refer to significant differences (*P* < 0.05).*

## Discussion

The objective of this study was to test the hypothesis that winter and spring night-warming upregulate the source and sink activities of winter wheat, which would be beneficial for grain yield formation. We observed that warming stimulated sink activity, which could therefore accelerate source carbohydrate depletion and, in turn, advance source activity (Tian et al., 2012). Sink formation (e.g., grain weight and grain number) and sink activity (e.g., grain-filling rate) enhance source productivity (e.g., photosynthesis) (Chen et al., 2014). Our results demonstrated that WW increased flag leaf area (**Figure 2**) and slightly increased specific leaf weight during the grain-filling stage (**Figures 2E,F**). In our previous study, we found that from the sowing to jointing stages and from the jointing to anthesis stages the wheat plants under WW and SW treatments showed higher growth rates (Fan et al., 2015). The mean night temperatures of the WW and ST treatment increased by 1.56–1.67°C and 1.78–1.92°C, respectively. Chen et al. (2014) indicated that increased night temperature can increase cell metabolism, the nighttime respiration rate of leaves and the growth rate of leaves. Under night-warming conditions, the number of leaf cells and plant metabolism become more vigorous. This may be the reason for the post-anthesis source improvement by night-warming, and this phenomenon benefited crop canopy light interception and contributed to higher grain weight.

Our previous study also showed that the growing degree days of the vegetative growth stage (from sowing to anthesis stage) increased by winter night-warming; thus, the pre-anthesis period was reduced (Fan et al., 2015). Winter wheat in the Yangtze River Basin of China, where the experiments were conducted, usually experiences extreme high-temperature weather during the grain-filling stage (Cao et al., 2011). The shortened pre-anthesis period caused by WW and SW may shift wheat plants in the post-anthesis period forward into a relatively cooler environment than the control (Liu et al., 2010). In addition, both WW and SW treatments increased the post-anthesis period of winter wheat, increasing the grain-filling time. Thus, wheat plants under WW and SW treatments benefited from complete physiological processes and grain-filling procedures. In the present study, WW significantly increased the post-anthesis flag leaf Pn and the maximum *A*_sat_ of Yangmai-13 and significantly increased the *V*_cmax_ of both cultivars (**Table 1**). Furthermore, the *J* of Yangmai-13 was increased by WW and SW (**Table 1**). Yamori et al. (2005) demonstrated that *V*_cmax_ is known to be enhanced with high-temperatures (the day/night air temperatures were 30/25°C for 1.5 months), while *J* could limit the photosynthesis rate under high-temperatures. Under warming treatments, the enhanced leaf PSII performance was due to the increased activities of photosynthesis-related enzymes and the enlargement of the leaf area (Martinez et al., 2014). These results indicate that moderate warming during the winter and spring improved the flag leaf photosynthetic capacity and Rubisco activity of winter wheat. Chlorophyll content during the grain-filling stage has been used as an efficient indicator of photosynthetic capacity in wheat cultivars (Christ and Hörtensteiner, 2014). Our study showed that WW and SW increased the chlorophyll content of flag leaves during the early stage of grain filling for both cultivars, while WW increased this chlorophyll content more than SW did (**Figure 3**). This finding indicates that winter night-warming enhanced photosynthesis in flag leaves by enhancing Rubisco carboxylation capacity, electron transport efficiency, ATP synthesis, and Calvin cycle processes. Consequently, our findings suggest that night-warming during winter could improve flag leaf morphology and photosynthetic capacities, which favor the grain-filling process of winter wheat.

Improved efficiency of photosystems also prevents the generation of ROS (Gallé et al., 2007). Wang et al. (2011) reported that pre-anthesis heat priming increases grain yield against subsequent high-temperatures during the grain-filling stage, and their finding was attributed to the improved photosynthetic and antioxidative activity in the acclimated plants. Our study showed that the WW treatment reduced post-anthesis ROS production (**Figures 4A–D**). This result indicates that plants under winter night-warming maintained a sufficient antioxidant capacity to avoid ROS accumulation. MDA content is used to evaluate the redox and osmotic adjustment status, which is important in the adaptation of plants to environmental stresses (Hung and Kao, 2004). Our study showed that the MDA content of flag leaves was lower for WW and SW than for NW for both cultivars after anthesis (**Figures 4E,F**). This indicates that WW and SW increased the capacity of the antioxidant system for scavenging ROS and regulating the accumulation of ROS. Similar results have been reported for *Kobresia pygmaea* (Yang et al., 2012) and *Betula pendula* (Riikonen et al., 2009). In addition, our study showed that WW and SW increased the SOD, POD, and CAT activity of flag leaves during the early grain-filling stage for both cultivars (**Figure 5**). This result suggests that the observed increase in SOD and POD activity would compensate for ROS accumulation. As CAT breaks down H_2_O_2_, an increase in CAT activity would have resulted in a reduction in H_2_O_2_, thereby attenuating oxidative damage of membrane. These data suggest that winter and spring night-warming increase post-anthesis antioxidant capacities to attenuate oxidative membrane damage, which is beneficial for improving flag leaf physiological activities.

To further illustrate the underlying mechanisms of increased post-anthesis photosynthesis and antioxidant properties induced by winter and spring night-warming, we analyzed the expression of photosynthesis-responsive and antioxidant enzyme-related genes. Wang et al. (2014) reported that Rubisco activase is a major limitation to photosynthetic capacity and a potential target for genetic manipulation that can enhance wheat productivity under high-temperatures. Kurek et al. (2007) reported that photosynthesis reduction is correlated with Rubisco deactivation resulted in Rubisco activase inhibition under high-temperature. In our study, *RcaB* expression in flag leaves was upregulated by WW (**Figure 6A**), and such a trend was associated with the enhanced photosynthetic capacity of flag leaves in WW plants compared with NW plants. Rampino et al. (2006) indicated that *Cab* was among the genes encoding a major chlorophyll a/b-binding polypeptide in the light-harvesting complex. Our study showed that the effects of WW and SW on *Cab* expression were not significant, except for Yangmai-13 under SW (**Figure 6B**). Furthermore, WW upregulated the expression of the antioxidant enzyme-related genes *Cu/Zn-SOD* (in Yangmai-13), *Mn-SOD* (in Yannong-19), and *CAT* (in Yangmai-13) compared with NW (**Figures 6C-E**). These higher antioxidant activities in the WW plants were associated with the upregulated expression of the antioxidant enzyme-related genes. The above evidence suggests that moderate night-warming during winter increases the expression of both photosynthesis-responsive and antioxidant enzyme-related genes, which directly regulate higher leaf photosynthesis and oxidation resistance abilities during the early grain-filling stage.

Our previous results showed that winter and spring night-warming increases the grain weight of both cultivars (Fan et al., 2015). Furthermore, we found similar positive effects of night-warming on the grain-filling rates during the early filling stage for both cultivars (**Table 2**), which suggests that winter and spring night-warming would benefit the grain filling of winter wheat. In our experiment, we used a moderate warming temperature of 1–2°C during the night, which is in line with the global warming rate projected by the Intergovernmental Panel on Climate Change (IPCC), resulting in the advancement of the phenophases of anthesis and maturity (Fan et al., 2015). This could shift the post-anthesis phase of winter wheat to more optimal temperature conditions for leaf photosynthesis and grain filling (Tian et al., 2012).

Our study showed that the grain sucrose content increased under WW (**Figure 7A**) and that the grain SS activity increased significantly under WW and SW for both cultivars (**Figure 7B**). Grain filling is mainly a process of starch biosynthesis and accumulation, and sucrose is the main substrate for the grain-filling process. SS activity is positively related to dry matter accumulation during grain filling, and SS activity is believed to be important in determining sink strength. Our study indicated that night-warming enhances sink strength and increases the amount of available sucrose for grain filling. CTK and IAA play important parts in regulating grain filling in cereals; the CTK content in rice spikelets is significantly positively correlated with grain development (Hao et al., 2009; Liu Y. et al., 2013). Xu et al. (2007) reported CTK and IAA regulate endosperm cell division in developing rice seeds. High IAA levels in a sink organ can create “attractive power,” leading to increased CTK levels in grains (Liu Y. et al., 2013). Our study showed that WW significantly increased the grain IAA and CTK concentrations at 14 DAA for both cultivars (**Figures 7C,D**). The present results indicate that IAA and CTK regulate grain filling of winter wheat in the early grain-filling stage, possibly due to the manipulation of endosperm cell division, thus creating sink strength.

Our study also showed that the increase in sink capacity was higher in Yangmai-13 (vernal type) than in Yannong-19 (semi-winter type) under WW. The optimal temperature during the vernalization phase is higher for the vernal type than for the semi-winter type of winter wheat (Singh et al., 2013). The warmer conditions under WW were more adaptive for Yangmai-13 growth and were particularly beneficial for spikelet and floret differentiation, which occurred during winter. We have previously reported that winter night-warming improves the pre-anthesis crop growth rate and enlarges the post-anthesis source of winter wheat, which is beneficial for grain dry matter accumulation during the grain-filling stage (Fan et al., 2015). Prolonged grain-filling duration certainly benefits grain formation. Consequently, the above evidence suggests that moderate nigh-warming during winter and spring would be beneficial to accelerate the grain-filling rate and enhance sink strength during the early grain-filling stage of winter wheat.

## Conclusion

We concluded from the results of the study that night-warming promoted source productivity by enlarging the flag leaf area and improving photosynthetic capacity. Night-warming also increased the antioxidant capacities, which attenuated oxidative membrane damage. This was seemingly controlled at the transcription level as indicated by the changes in the expression of both photosynthesis-responsive and antioxidant enzyme-related genes. Night-warming enhanced sink strength and accelerated the grain-filling rate; however, the increases were greater during winter night-warming than during spring night-warming. The above evidence demonstrates that winter night-warming improves grain filling and the flag leaf physiological characteristics of winter wheat, resulting in an improved performance of wheat crop.

## Author Contributions

YF, TD, ZH, and ZT designed the experiment. YF conducted the study, collected and analyzed the data, and prepared the draft. YY and CH helped in sampling and the measurements of physiological parameters. DJ and CM helped in drafting the manuscript and the interpretation of the results. MA helped in revising the manuscript and the responses to the comments.

## Conflict of Interest Statement

The authors declare that the research was conducted in the absence of any commercial or financial relationships that could be construed as a potential conflict of interest.
